# Investigating the genetic architecture of eye colour in a Canadian cohort

**DOI:** 10.1016/j.isci.2022.104485

**Published:** 2022-05-30

**Authors:** Frida Lona-Durazo, Rohit Thakur, Erola Pairo-Castineira, Karen Funderburk, Tongwu Zhang, Michael A. Kovacs, Jiyeon Choi, Ian J. Jackson, Kevin M. Brown, Esteban J. Parra

**Affiliations:** 1 Department of Anthropology, University of Toronto at Mississauga, Mississauga, ON, Canada; 2 Laboratory of Translational Genomics, Division of Cancer Epidemiology and Genetics, National Cancer Institute, National Institutes of Health, Bethesda, MD, USA; 3 Integrative Tumor Epidemiology Branch, Division of Cancer Epidemiology and Genetics, National Cancer Institute, National Institutes of Health, Bethesda, MD, USA; 4 MRC Human Genetics Unit, Institute of Genetics and Cancer, University of Edinburgh, UK; 5 Roslin Institute, University of Edinburgh, Easter Bush, Midlothian, UK

**Keywords:** Genetics, Genomics, Human Genetics

## Abstract

Eye color is highly variable in populations with European ancestry, ranging from low to high quantities of melanin in the iris. Polymorphisms in the *HERC2/OCA2* locus have the largest effect on eye color in these populations, although other genomic regions also influence eye color. We performed genome-wide association studies of eye color in a Canadian cohort of European ancestry (N = 5,641) and investigated candidate causal variants. We uncovered several candidate causal signals in the *HERC2/OCA2* region, whereas other loci likely harbor a single causal signal. We observed colocalization of eye color signals with the expression or methylation profiles of cultured primary melanocytes. Genetic correlations of eye and hair color suggest high genome-wide pleiotropy, but locus-level differences in the genetic architecture of both traits. Overall, we provide a better picture of the polymorphisms underpinning eye color variation, which may be a consequence of specific molecular processes in the iris melanocytes.

## Highlights

• Genome-wide association studies of eye color in 5,641 participants
• Multiple independent candidate causal variants were identified across *HERC2/OCA2*
• Single candidate causal variants observed on or near *IRF4, SLC24A4, TYR,* and *TYRP1*
• Colocalization of eye color signals with expression and methylation profiles

## Graphical abstract

**Figure:**
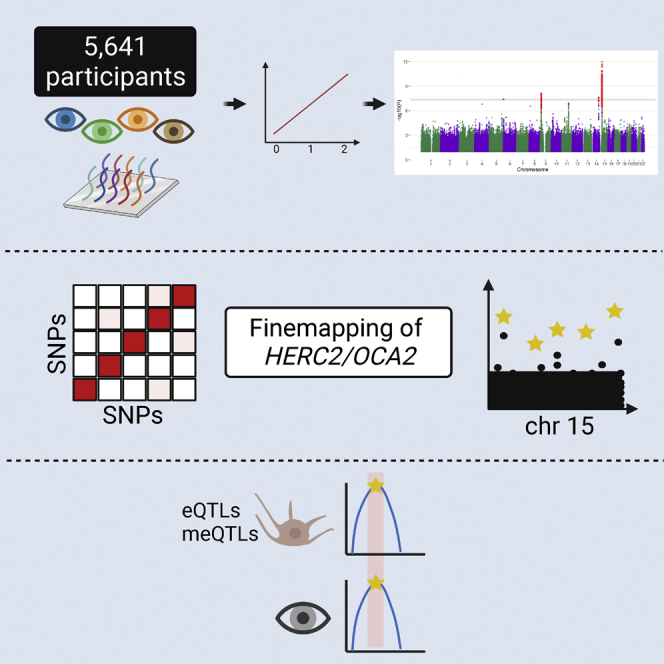

## Introduction

Pigmentation levels in the iris vary among humans, ultimately leading to different eye colors. The melanin pigment in the iris is synthesized in the melanocytes, within organelles named melanosomes (Sturm and Frudakis, 2004). Eye color diversity is a consequence of different amounts of melanin concentrated in the melanocytes of the iris. In addition, the shape and distribution of melanosomes influence eye color variation. The mechanism is different from that of hair and skin pigmentation, in which two types of cells, melanocytes and keratinocytes (i.e. the epidermal melanin unit), play a key role in the production and distribution of melanin to give hair and skin color (Rees, 2003; Lin and Fisher, 2007; Parra, 2007). In addition, out of the two types of melanin synthesized by melanocytes (i.e. eumelanin, a brown/black pigment and pheomelanin, an orange/yellow pigment), different categorical iris colors are a result of variation mainly on eumelanin content, whereas there is little, nonsignificant variation on pheomelanin quantity, based on measurements on cultured uveal melanocytes (Wakamatsu et al., 2007).

At a molecular level, blue irises appear as melanin-free melanocytes, in which molecules in the iris scatter short blue wavelengths to the surface (Sturm and Frudakis, 2004). Green irises have medium levels of eumelanin, whereas high levels of eumelanin result in brown irises. Therefore, broad eye color classifications (i.e. blue, green, hazel, brown) cover a continuum of low to high quantities of eumelanin accumulated in the iris (Sturm and Frudakis, 2004).

Twin studies have shown that eye color is a highly heritable trait (>85%) and that it does not significantly vary throughout an adult’s lifespan (Bito et al., 1997; Larsson et al., 2003). Furthermore, association studies have demonstrated that eye color has a polygenic architecture (Sulem et al., 2007; Edwards et al., 2016; Lloyd-Jones et al., 2016; Adhikari et al., 2019). Some of the loci with moderate/large effects associated with eye color variation are at or near the following genes: *OCA2, TYR, TYRP1, SLC45A2, SLC24A4, SLC24A5,* and *IRF4*. However, the variant with the largest effect on eye color variation is an intronic SNP (rs12913832) located in an enhancer within the gene *HERC2* that regulates the expression of the downstream gene *OCA2* (Visser et al., 2012). Functional studies have shown that the A-allele of rs12913832 allows the formation of a chromatin loop with the promoter of *OCA2*, facilitating the transcription of the gene. In contrast, the G-allele hinders the formation of the chromatin loop, leading to a diminished expression of *OCA2* (Visser et al., 2012; Visser et al., 2014).

The SNP rs12913832 is the key regulatory element of *OCA2*. But it has been hypothesized that additional distal elements within the same region may be involved in the regulation of *OCA2,* a process that often is tissue specific (Palstra, 2009; Visser et al., 2014). In fact, through conditional analyses of association, genome-wide association studies (GWAS) have highlighted the presence of additional SNPs associated with variation in pigmentary traits (i.e. skin, hair, and eye pigmentation) within the *HERC2/OCA2* region (Beleza et al., 2013; Adhikari et al., 2019; Lona-Durazo et al., 2019; Landi et al., 2020).

These studies have identified variants within *HERC2* and *OCA2* that are in low (r^2^ < 0.2) linkage disequilibrium (LD) with rs12913832 (e.g. rs4778249, rs1667392, rs4778219, rs1800407, rs1448484), as well as other distant candidate regulatory variants near or within the *APBA2* gene (e.g. rs4424881, rs36194177), which is located ∼700kb away from *OCA2*. However, pinpointing additional causal variants within the *HERC2/OCA2* region is challenging due to the complex LD patterns among the genetic variants and the lack of tissue-specific regulatory annotations. For instance, the Gene and Tissue Expression (GTEx) database (Consortium et al., 2017) includes skin tissue, which beyond a very small proportion of melanocytes, encompasses a diverse set of cell types not involved in pigmentation variation.

In order to improve our understanding of the genetic mechanisms behind eye pigmentation and melanocyte biology, in this paper we present the results of a GWAS of eye color conducted in a Canadian cohort from the Canadian Partnership of Tomorrow’s Health (CanPath), along with fine-mapping analyses. We combined these results with gene expression and methylation data of cultured melanocytes by conducting colocalization analyses and transcriptome-wide association studies (TWAS). Our main results indicate that there are several candidate signals in the *HERC2/OCA2* region associated with eye color, a different pattern from what is observed for hair color in the same sampled population. By integrating expression and methylation data assayed in melanocytes, we gain a better picture about how genetic polymorphisms may modulate eye color variation.

## Results

### Eye color distribution in the CanPath cohort

**Figure 1:**
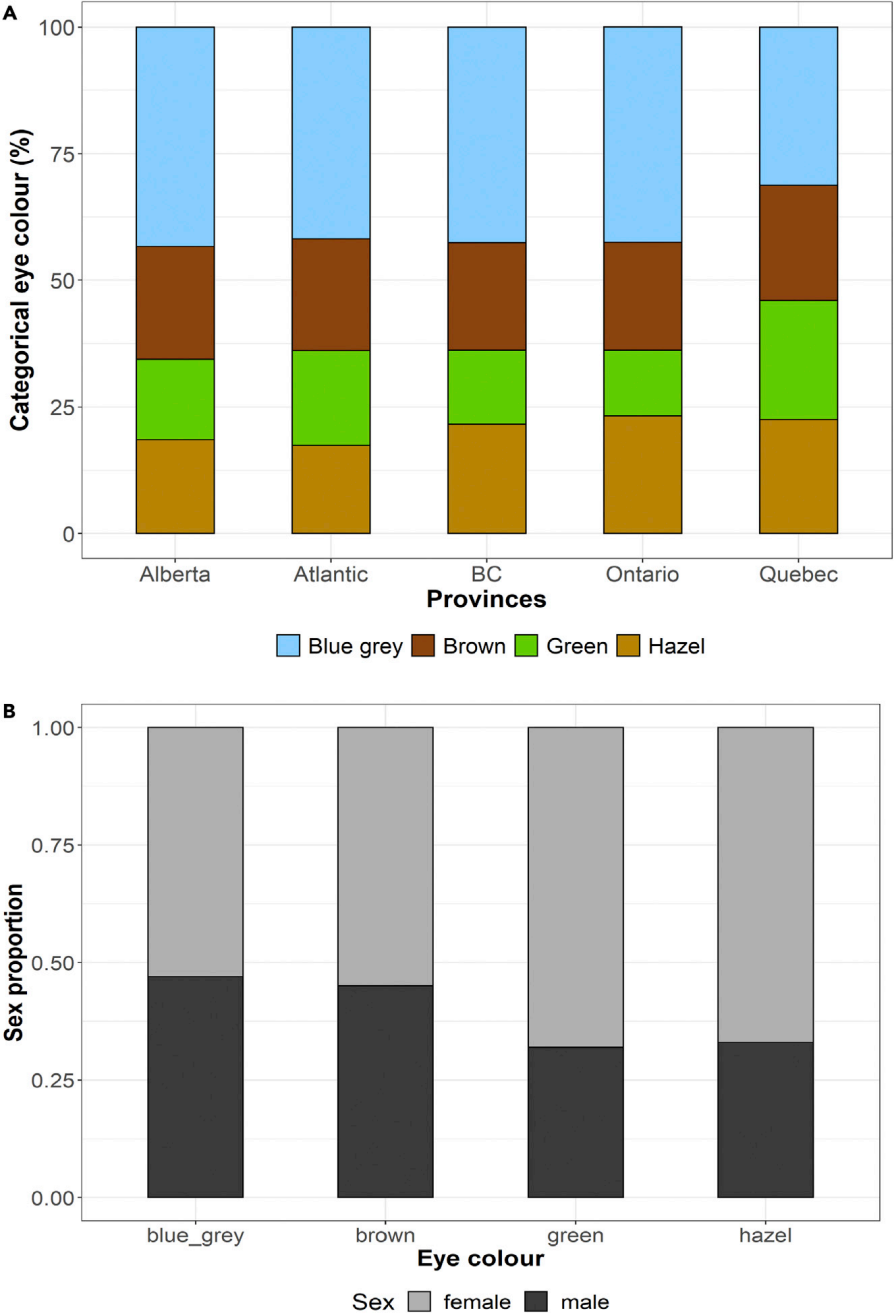
Distribution of eye color categories in the CanPath (A) Percentage of the eye color categories stratified by province. (B) Proportion of sexes across eye color categories.

A total of 5,732 participants of the Canadian Partnership for Tomorrow’s Health (CanPath), who were genotyped using two genome-wide genotyping arrays (See STAR Methods for details), also self-reported their natural eye color using one of six possible answers: blue, gray, green, amber, hazel, or brown. We excluded amber eye color individuals due to the low number of individuals who self-reported this category. After quality control of the genotypes (i.e. exclusion of poor-quality samples and PCA outliers), we kept 5,641 individuals for further analyses. Overall, the distribution of eye color categories was quite similar across all provinces sampled (Figure 1), in which green and hazel were the least frequent categories and blue was the most common one. An important exception is the significantly lower proportion of individuals who self-reported blue eye color in Quebec, compared with other provinces (chi-square test: 104.39, df = 4, p value < 0.01). This pattern may be explained by the high proportion of French ancestry in the Quebec population (See Figure S1) due to the migration and settlement of French people in the province relatively recently (Bherer et al., 2011). This hypothesis is supported by the difference in allele frequencies between Quebec and all other provinces, in which the *HERC2* rs12913832 G-allele has a lower frequency than in other provinces (see Table S1). In addition, a higher proportion of females self-reported green and hazel eye colors, relative to their male counterparts (chi-square test for green and hazel combined = 244.49; df = 1; p value < 0.01), which is similar to the observations previously reported in the case of green eye color (Sulem et al., 2007). Compared with several reported eye color frequencies per country, the CanPath eye color distributions differ from those found in other European and Asian countries, with the blue eye color frequency being most similar to that of Germany (39.6%) (Katsara and Nothnagel, 2019).

### Genome-wide association studies and meta-analyses

We performed GWAS of eye color on each genotyping array (genotyped and imputed single-nucleotide polymorphisms (SNPs)) using a linear mixed model and an additive genetic model, using GCTA 1.26.0 (Yang et al., 2011, 2014). We coded eye color categories as follows: 1 = blue or gray, 2 = green, 3 = hazel, and 4 = brown. We included sex, age, and the first ten principal components (PCs) as fixed effects and a genetic relationship matrix (GRM) as random effect to control for subtle population structure. We did not detect residual population substructure, based on Q-Q plots, in which observed p values did not show an early deviation from the expected p values (See Figure S2).

**Figure 2:**
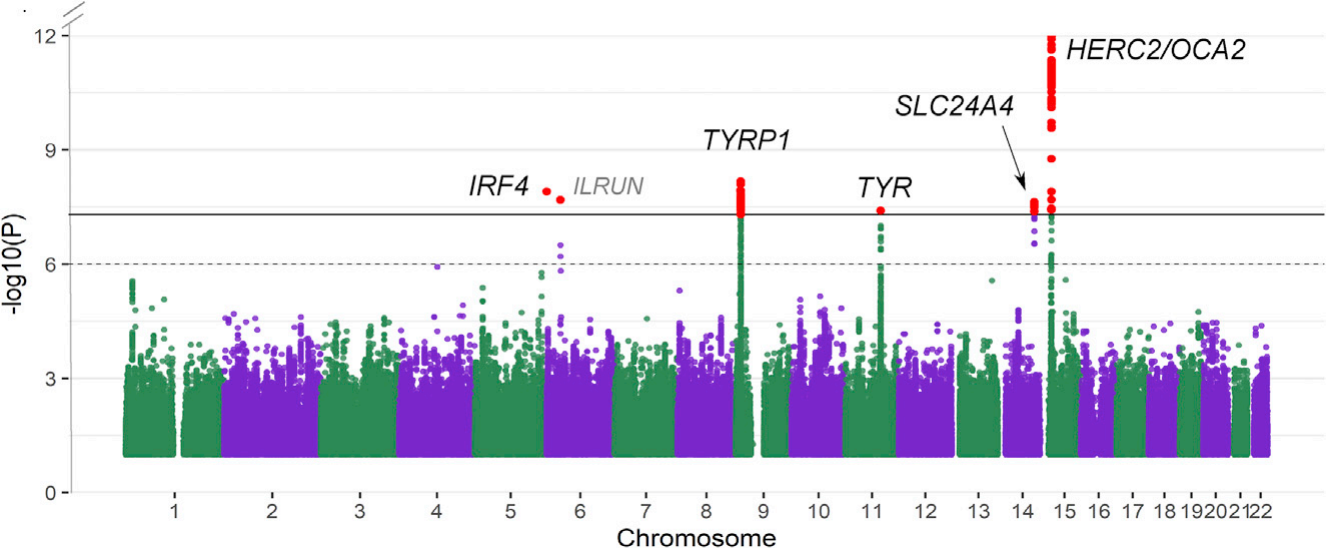
Manhattan plot of eye color meta-analysis based on a linear mixed model The dotted line indicates the suggestive threshold (p = 1e-06), and the continuous line denotes the genome-wide threshold (p = 5e-08). The Y axis has been limited to truncate strong signals at the locus in chromosome 15. The full figure is available as Figure S4.

We then carried out a meta-analysis using the summary statistics (beta and SE) including the two GWAS on METASOFT v2.0.1 (Han and Eskin, 2011). Q-Q plots of the meta-analyses (See Figure S3) and LD Score regression (intercept = 0.9935) indicated no residual population structure. We identified several known genome-wide significant loci (p value ≤ 5e-08) associated with eye color (Figure 2; see Figure S4), overlapping or near the genes *TYRP1* (lead SNP: rs1326779; beta = 0.139; SE = 0.024)*, IRF4* (lead SNP: rs12203592; beta = −0.164; SE = 0.029), *TYR* (lead SNP: rs1126809; beta = −0.136; SE = 0.024), *SLC24A4* (lead SNP: rs4144266; beta = −0.124; SE = 0.022), and *HERC2* (lead SNP: rs1129038; beta = −1.239; SE = 0.024). In addition, we observed a signal on chromosome 6 overlapping the *ILRUN* gene (lead SNP: rs116072038; beta = −0.438; SE = 0.077), a locus that has not been previously associated with pigmentation. Data S1 summarizes the suggestive and genome-wide associated SNPs.

### Fine-mapping of GWAS hits

We conducted approximate conditional and joint analyses of association using GCTA-COJO (Yang et al., 2012), and using as LD reference the samples genotyped in this study with the UKBB array, to investigate if the genome-wide significant loci were being driven by one or more independent signals.

On the *IRF4*, *TYRP1, TYR,* and *SLC24A4* regions, we identified one independent genome-wide significant SNP per locus (see Table S3), corresponding to known causal variants, such as rs12203592 on *IRF4* and rs1126809 on *TYR*. The lead SNP on *SLC24A4* is in high LD (r^2^ = 0.98) with rs12896399, a SNP previously associated with pigmentation (Sulem et al., 2007; Eriksson et al., 2010). In the case of *TYRP1*, the selected SNP (rs1326779) is downstream of *TYRP1* and has not been previously highlighted in eye color studies. On the *HERC2/OCA2* region, we identified six independent SNPs overlapping *OCA2* and *HERC2* with p values in the conditional analysis exceeding the genome-wide significant threshold (see Table S3). Several of these SNPs have evidence of heterogeneity among the two studies, as indicated by I^2^ and Cochran’s Q values in the meta-analysis. However, they all have genome-wide significant p values in the random effects (RE2) model too, which takes into account heterogeneity among studies (see Table S3). To validate our results, we carried out the same GCTA-COJO analysis a second time, using samples genotyped with the GSA array as LD reference. We obtained concordant results, with multiple independent SNPs on the *HERC2/OCA2* region and a single SNP highlighted on the other pigmentation associated loci (i.e. *IRF4, TYR, SLC24A4,* and *TYRP1*) (see Table S4).

We also carried out a Bayesian fine-mapping analysis, in which all possible combinations of SNPs are iteratively considered without arbitrary selection of conditioned SNPs. We used the program FINEMAP (Benner et al., 2016) to perform fine-mapping analysis and to identify candidate causal SNPs for functional prioritization. In agreement with the GCTA-COJO analysis, by using FINEMAP we identified known pigmentation loci harboring one causal signal within *IRF4*, *TYR, SLC24A4,* and *TYR* (Data S2). On the *IRF4* locus, the only candidate causal SNP with considerable evidence of causality (log_10_BF > 2) was the same SNP highlighted by GCTA-COJO (rs12203592; PIP = 0.999). In contrast, the missense SNP rs1126809 on *TYR* had a low posterior inclusion probability (PIP = 0.209), due to high LD with other nearby SNPs. Other candidate causal SNPs in the 95% credible set of the *TYR* locus include intergenic variants and one SNP (rs11018578) on the 3′UTR region of *NOX4*. On the *SLC24A4* locus, the variants with considerable evidence of causality (i.e. log10BF ≥ 2) include intronic SNPs within *SLC24A4* and other variants upstream of the gene, including the SNP rs12896399 (log_10_BF = 2.58) (Data S2).

On the *TYRP1* locus, all candidate causal SNPs in the credible set had a low (<0.1) PIP, most likely due to high LD among multiple SNPs in the locus (See Figure S5). The 95% credible set includes rs10809826 and rs1408799, two SNPs that have been previously associated with eye color (Sulem et al., 2008; Zhang et al., 2013; Galván-Femenía et al., 2018; Adhikari et al., 2019). Among the SNPs in the same 95% credible set, rs13297008 is located upstream of *TYRP1*, and it overlaps a DNase Hypersensitive Site identified in foreskin melanocytes (Data S2), indicative of an active transcriptional regulator region. We did not observe any poorly imputed or unimputed variants overlapping regulatory regions found in melanocytes. However, we did find that rs13297008, rs2733831, and rs13296454 are in an active transcription start site (TSS) state on foreskin melanocytes only (across the tissues tested with the 15-core chromatin states). These SNPs are suggestive of either association or genome-wide significance, and all three are within the 95% credible set (Data S1).

### Multiple *HERC2/OCA2* variants associated with eye color variation

**Figure 3:**
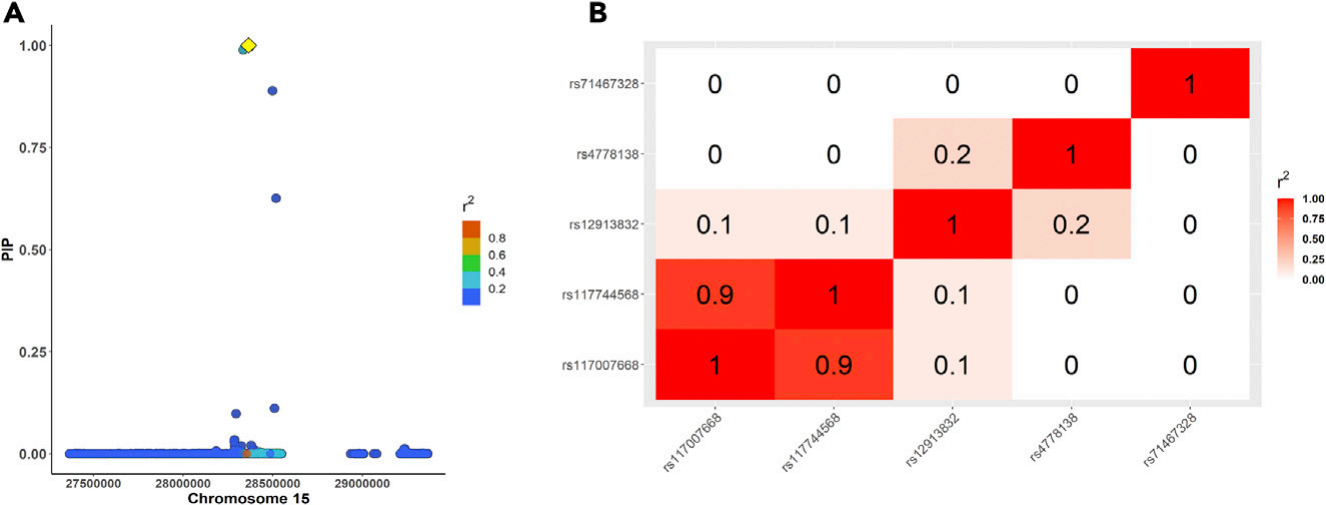
Fine-mapping of the *HERC2/OCA2* locus (chromosome 15) associated with eye color (A) FINEMAP regional plot of the posterior inclusion probability (PIP), in which the lead SNP (rs12913832) is highlighted in yellow and LD (r^2^) correlations are shown in respect to the lead SNP. (B) Matrix of LD correlations among the SNPs with the highest PIP on each of the five 95% credible sets.

By applying a Bayesian fine-mapping approach on the *HERC2/OCA2* region, we identified five candidate causal signals (i.e. five 95% credible sets) associated with eye color. Within these signals, three SNPs had a PIP >0.98 (Table 1 and Figure 3A). These results suggest independent causality of various signals in the locus. One of the candidate SNPs within *HERC2* is rs12913832, a known enhancer that regulates the expression of *OCA2* (Visser et al., 2012). In addition, three other SNPs within *HERC2* and one within *OCA2* were nominated as candidate causal loci, all of which fall within introns. These results are similar to the conditional analysis with GCTA-COJO, in which the independent SNPs in the locus encompass both *OCA2* and *HERC2*, but the selected SNPs do not fully overlap. Importantly, all Bayesian fine-mapped SNPs in the locus had genome-wide significant p values on each sample and the same direction of effect. We annotated the putative regulatory function of the SNPs in all five credible sets using diverse databases (e.g. ENCODE, Roadmap Epigenomics Project). Aside from the overlap of rs12913832 (*HERC2*) with an open chromatin region in foreskin melanocytes, only the SNP rs117007668 is located within an open chromatin region in foreskin melanocytes (Data S2).

**Table 1:** Summary statistics of the candidate causal SNPs with log_10_BF ≥ 2 in the *OCA2/HERC2* region on chromosome 15, associated with eye color

Credible Set	rsid	Position in chr 15	Gene	Beta	SE	p value (FE)	p value (RE2)	MAF	I^2^	Cochran’s Q	Cochran’s Q p value	PIP	Log_10_BF
1	rs12913832	28365618	*HERC2*	−1.26	0.02	0	0	0.23	96.25	26.66	243E-07	1.00	13.14
2	rs117007668	28371422	*HERC2*	0.99	0.08	5.43E-38	8.30E-38	0.01	81.03	5.27	0.02	1.00	5.59
3	rs4778138	28335820	*OCA2*	0.81	0.03	2.56E-162	1.51E-161	0.13	0.00	0.25	0.62	0.99	5.08
4	rs117744568	28498692	*HERC2*	0.87	0.06	3.95E-46	1.01E-45	0.02	73.99	3.85	0.05	0.89	4.04
4	rs117743506	28510460	*HERC2*	0.85	0.06	6.46E-46	1.96E-45	0.02	65.92	2.93	0.09	0.11	2.23
5	rs71467328	28518229	*HERC2*	−0.48	0.05	5.60E-18	1.13E-17	0.04	27.37	1.38	0.24	0.63	3.36
5	rs1597196	28294922	*OCA2*	0.42	0.03	3.86E-55	1.27E-54	0.18	63.25	2.72	0.10	0.10	2.17

FE = fixed-effects model; RE2 = random-effects model; MAF = minor allele frequency; I^2^ and Cochran’s Q: meta-analysis heterogeneity indices; PIP = posterior inclusion probability; log_10_BF _=_ log_10_ of Bayes Factor.

We then explored the LD patterns among the candidate causal variants (Table 1) in the credible sets using the CanPath genotypes (i.e. the same LD matrix used for fine-mapping) and considering the genotype probabilities, computed with LDStore v2.0 (Benner et al., 2017). Among the top candidate causal variants across the five credible sets (Table 1), most correlations are low (r^2^ ≤ 0.2), with the exception of rs117007668 and rs117744568, which are in high LD (r^2^ = 0.9) (Figure 3B). We further compared D′ values among these same SNPs computed on LDLink, using as a proxy the European populations of the 1000 Genomes Project (Machiela and Chanock, 2015). The D′ patterns, compared with r^2^ values, reflect the allele frequency differences among the SNPs (D’ > 0.6 in most cases) and suggest that the candidate causal SNPs are not in complete linkage equilibrium (See Figure S6).

We explored with HaploReg (version 4) (Ward and Kellis, 2012, 2016) if other variants in the same LD block as our credible set SNPs in the *OCA2/HERC2* locus harbor a putative regulatory function, to consider genetic variants that may have not been present in our dataset after imputation. By using as input the top SNP on each of the five credible sets (Table 1), we identified a nominally significant enrichment of enhancers (as defined by the 15-state core ChromHMM model) in foreskin melanocytes (binomial test compared with all 1KGP variants with MAF ≥5%; p value = 0.0169). The SNP rs117743506, which is in high LD with rs117744568 and in the same credible set, has an enhancer state in foreskin melanocytes. In addition, it may alter the motif of POU3F2, a transcription factor (TF) present in melanoma cell lines known to alter the expression of pigmentation genes (i.e. *MITF, KITLG*), although a recent study suggests that this TF does not have a role in normal skin melanocytes (Larue et al., 2010; Chitsazan et al., 2020).

Finally, we explored SNP-SNP interactions across the top GWAS SNPs that passed the genome-wide significant threshold using the program CASSI and included also the loci fine mapped in the *OCA2/HERC2* region (SNPs in Figure 3). By applying a Bonferroni-corrected p value threshold = 0.0015 (i.e. 0.05/33 pairwise tests), we did not identify significant interactions. However, there were nominally significant interactions between *SLC24A4* (index SNP: rs4144266) and *HERC2/OCA2* (SNPs: rs12913832 and rs4778138) (see Table S7).

### Associations with eye color in recent studies

By investigating eye color variation in the *OCA2* locus, Andersen et al. (2016) identified that two missense SNPs within *OCA2* (rs121918166 and rs74653330) have a measurable effect on eye color variation, in which the alternative alleles decrease melanin levels, even in a heterozygote state. These two SNPs were rare in our sample (MAF <1%); therefore, we did not consider them in our GWAS analyses. By looking at the genotype-level data in these two rare SNPs, we identified 108 individuals heterozygous for either rs121918166 or rs74653330. Among these, 16 individuals were homozygous for the nonblue eye color rs12913832-AA genotype, but none self-reported having blue eye color (brown = 11; green = 1; hazel = 4). In addition, among heterozygous individuals for either one of the rare SNPs (i.e. rs121918166 or rs74653330), 12 individuals were also heterozygous for the nonblue eye color rs12913832-AG genotype and self-reported blue eye color. These results indicate that these two rare variants do not account for the incidences of blue eye color with an rs12913832-AA background in the CanPath, but they may influence the eye color phenotype under an rs12913832-AG background. Furthermore, we identified in our sample a subset of individuals (N = 904) harboring the rs12913832:GG genotype, 21 of whom self-reported brown eye color, 631 green eye color, and 252 hazel eye color, suggesting that the rs12913832:GG genotype does not exclusively yield a blue eye color.

We compared the *OCA2/HERC2* haplotypes previously described for eye color in European ancestry populations, with the fine-mapped loci reported in the present study. Donnelly et al. (2012) identified three haplotypes in the region, two of which match the fine-mapped SNPs we have identified: rs12913832 (BEH2) and rs4778138 (BEH1). The third haplotype includes rs916977 and rs1667394, but these two SNPs are not highly correlated (i.e., r^2^ < 0.6) with any of our fine-mapped loci. The IrisPlex System is widely used to predict blue/brown eye color based on common genetic polymorphisms (Walsh et al., 2012). Currently, it includes two SNPs in the *OCA2/HERC2* region (rs12913832 and rs1800407). The rs1800407 SNP is a missense SNP in *OCA2,* known to be involved in pigmentation variation (Donnelly et al., 2012; Pospiech et al., 2014; Kidd et al., 2020). This SNP did not pass the quality control in our study, but other SNPs in high LD are not within the fine-mapped 95% credible sets. In addition, other common SNPs across other genes are also included in the IrisPlex System, namely *SLC24A4* (rs12896399), *SLC45A2* (rs16891982), *TYR* (rs1393350), and *IRF4* (rs12203592). Importantly, in our study there is a genome-wide significant association of *TYRP1* with eye color, but this locus is not included in the IrisPlex System.

Adhikari et al. (2019) conducted conditional analyses of association of eye color (measured qualitatively and quantitatively) in Latin American individuals with mainly European and Native American ancestry and identified up to five independent signals in the *HERC2/OCA2* locus (indexed by: rs4778219, rs1800407, rs1800404, rs12913832, and rs4778249). Aside from rs12913832, none of their index SNPs are within the candidate causal SNP sets in our sample, even though rs1800404 was genome-wide significant in our meta-analysis (p value = 1.18 × 10^−11^). In addition, they identified three novel loci associated with eye pigmentation: *DSTYK* (chromosome 1)*, WFDC5* (chromosome 20), and *MPST* (chromosome 22). We followed-up each of the three index SNPs in our meta-analyses (rs3795556, rs17422688 and rs5756492), but failed to replicate the former two SNPs using a Bonferroni correction (p value threshold = 0.005), whereas rs5756492 was not present in our meta-analysis. These differences may be driven by population ancestry differences, given that the CANDELA cohort includes recently admixed individuals from Latin America, although the phenotyping approach may also be driving these differences.

Finally, the largest eye color GWAS to date conducted in populations of mainly European ancestry reported several novel loci, which had not been previously associated with eye color, and a subset of them has not been previously associated with any pigmentation trait (eye, hair, or skin pigmentation) (Simcoe et al., 2021). However, the *ILRUN* locus identified in the present study was not among their novel signals, nor we were able to replicate it. In addition, they conducted conditional analyses of association to identify secondary signals on the significant loci, in which they identified a total of 115 independent signals, including three signals in chromosome X. Notably, they identified 33 independent signals in the *HERC2/OCA2* region and two signals in the nearby gene *GABRB3*. We followed-up their signals (Table S1 from Simcoe et al., 2021) in our meta-analyses and identified 50 SNPs nominally significant in our meta-analysis (p value ≤ 0.05), all with a consistent direction of effect between both studies (see Table S5). This set of SNPs includes novel associations with eye color and/or pigmentation traits included in their study (i.e. *DTL, MITF, PDCD6/AHRR, ADRB2, GCNT2,* and *SIK1*). After considering a Bonferroni correction (0.05/112; p value ≤ 4.46e-04), 16 SNPs remained significant, all of which overlap known pigmentation genes (*IRF4, TYRP1, TYR, OCA2,* and *HERC2*).

Lastly, we checked if the independent SNPs associated with eye color identified with GCTA-COJO by Simcoe et al. (2021) overlap with our eye color SNPs highlighted by GCTA-COJO (See Table S3). We identified an overlap of three SNPs: rs12203592 (*IRF4*), rs1126809 (*TYR*), and rs1129038 (*HERC2*). Interestingly, even though Simcoe et al. identified several independent loci in the *OCA2/HERC2* region, the known rs12913832 SNP is not among them, likely because another SNP in perfect LD has a lower p value, which is similar to what we observe in our GCTA-COJO analysis (i.e. rs1129038). We also compared the same independent SNPs identified by Simcoe et al. with our candidate causal loci as defined by FINEMAP and found three overlapped SNPs: rs12203592 (*IRF4*), rs1126809 (*TYR*), and rs13297008 (*TYRP1*).

### Colocalization with expression and methylation QTLs from cultured melanocytes

We performed colocalization analyses with hyprcoloc (Foley et al., 2021) of the eye color meta-analysis with melanocyte gene expression and methylation *cis*-QTLs (eQTLs, meQTLs, respectively) to explore if there were shared causal signals (see STAR Methods for details). Through the colocalization of GWAS with eQTLs, we identified a region overlapping *OCA2* and AC090696.2 (the latter being a transcript that partially overlaps *OCA2*) (Table 2), in which the candidate marker is rs12913832. We also colocalized meQTLs with GWAS hits (Table 2) overlapping the gene body of *HERC2* (tagged by cg25622125, cg27374167, and cg05271345) using a posterior probability threshold of 0.8. Notably, we did not find colocalized eQTLs on the *TYRP1* locus that passed the probability cutoff.

**Table 2:** Colocalization results of expression and methylation QTLs (eQTL and meQTL, respectively) with GWAS eye color SNPs, showing colocalized SNPs with a posterior probability ≥0.8

Chromosome	Candidate SNP	Posterior probability	Regional probability	Posterior explained by SNP	Gene/Methylation annotation	QTL
15	rs12913832	0.9999	1	1	*OCA2*	eQTL
15	rs12913832	0.9864	0.9891	1	AC090696.2	eQTL
15	rs12913832	0.9991	0.9995	1	*HERC2* (Body) | OpenSea	meQTL
15	rs12913832	0.9855	0.9872	1	*HERC2* (Body) |S_Shelf	meQTL
15	rs12913832	0.9807	0.9831	1	*HERC2* (Body) |S_Shore	meQTL

The methylation annotation indicates the location with respect to the nearest gene (TSS. = transcription start site), as well as the location of the tagged CpG marker within the CpG island.

### Transcriptome-wide association studies

We conducted TWAS using a subset of the CanPath cohort as LD reference and the expression weights from cultured melanocytes to predict the gene expression profile with FUSION (Gusev et al., 2016). Our results highlighted the expression of three genes as significantly associated with eye color: *OCA2*, *SLC24A4*, and *RIN3* (See Table S6; See Figure S7). The gene *RIN3* is located near *SLC24A4*, and by conducting conditional TWAS we have shown that these two genes are not independent from each other (See Figure S8).

### Genetic correlations

We calculated the genetic correlation between eye and hair color using the data from the two CanPath genotyping arrays for which we had full phenotype data (Lona-Durazo et al., 2021). Using a linear scale for both traits and the same covariates as used in the GWAS (See STAR Methods for details), there is a genetic correlation (r_g_) of 55% (SE = 0.12; p value = 7.33e-6) and 69% (SE = 0.21; p value = 0.001) on the UK Biobank and GSA arrays, respectively. Similar to the approach used in a previous study (Lin et al., 2016), we then calculated the genetic correlation without controlling for the effect of significant principal components and obtained genetic correlation values of 63% (SE = 0.08; p value = 3.41e-15) and 79% (SE = 0.15; p value = 1.39e-07) on the UK Biobank and GSA arrays, respectively. These results are in line with the genetic correlations previously reported (Lin et al., 2016), in which they found a lower correlation when including principal components as covariates, due to the correlations between ancestry captured by the PCs and eye or hair color. Nevertheless, by not controlling for significant PCs, we may as well be capturing ancestry in the genetic correlation estimation.

## Discussion

In this paper, we present the results of our genome-wide association studies of eye color, as measured categorically through self-reports, from 5,641 participants of the Canadian Partnership for Tomorrow’s Health (CanPath). We did not identify new loci associated with eye color that were successfully replicated, and we focused on performing downstream analysis to pinpoint candidate causal SNPs, specifically on those loci for which a functional variant has not yet been identified or in which there is evidence of more than one independent signal. We found that fine-mapping provides evidence for multiple independent SNPs within the *HERC2/OCA2* region, whereas other loci likely have a single causal signal. Furthermore, we characterized our GWAS signals by using colocalization analyses with expression and methylation QTLs of cultured melanocytes and conducted TWAS, in which we identified the expression of *SLC24A4/RIN3* and *OCA2* as significantly associated with eye color. Lastly, we explored the genetic correlations between hair and eye color in the CanPath cohort.

One of the caveats of this study is that we utilized eye color categories self-reported by participants of the CanPath cohorts, as categorical classifications do not capture as well iris color variation as quantitative measures (Liu et al., 2010; Norton et al., 2015; Edwards et al., 2016). In our sample we have identified significant differences in self-reporting of eye color between sexes, and although these may be a reflection of true sex differences, as has been previously reported in other studies reporting self-assessed categorical eye color (Sulem et al., 2007), we cannot discard the possibility of a self-reporting bias. This limitation is counter-balanced by the relatively large sample size, in comparison to the majority of previous studies (Kayser et al., 2008; Candille et al., 2012; Beleza et al., 2013), with the exception of the largest recent GWAS of eye color (Simcoe et al., 2021). Furthermore, significantly associated loci from self-reported eye color are useful in forensics, for predicting eye color categories, which the human eye can easily distinguish. Indeed, we have here identified most signals associated with eye color that are used in the IrisPlex eye color prediction system (Walsh et al., 2014, 2017; Chaitanya et al., 2018). One of the loci not included currently in the IrisPlex system is *TYRP1,* a locus that could potentially improve the eye color prediction system. However, it is important to point out that structural features of the iris (i.e. contraction furrows, Wolfflin nodules, heterochromia) also contribute to color perceptions, but we are not able to distinguish them using the current dataset.

Through our GWAS meta-analysis we identified five known loci associated with eye color, encompassing the genes *SLC24A4, IRF4, TYRP1, TYR,* and *HERC2/OCA2*, similar to what was identified in a recent large GWAS of both categorical and quantitative eye color loci in a Latin American (CANDELA) cohort (Adhikari et al., 2019). The only two known pigmentation loci that our GWAS failed to identify as significantly associated with eye color encompass the genes *SLC24A5* on chromosome 15 and *SLC45A2* on chromosome 5, in which missense variants (rs1426654 and rs16891982) are known to alter pigmentation traits (Lamason et al., 2005). The missense SNP on *SLC24A5* was rare in our sample (MAF <1%) hence excluded, in line with frequencies observed in the 1000 Genomes Project European populations, in which the alternative allele is nearly fixed. In the case of the *SLC45A2* locus, the missense SNP did not reach genome-wide significance (p value = 1.71e-6).

Our fine-mapping analyses identified known causal pigmentation loci in the credible sets, such as rs12203592 on *IRF4*, rs1126809 on *TYR*, and rs12913832 on *HERC2*. Contrary to what has been observed for hair pigmentation (Adhikari et al., 2019), we identified here one independent SNP in the *TYR* locus associated with eye color, even though there is at least another independent missense variant (rs1042602) known to alter melanin synthesis within the same gene, in addition to candidate regulatory variants in the upstream *GRM5* gene associated with skin pigmentation (Stokowski et al., 2007; Sulem et al., 2007; Liu et al., 2010, 2015; Beleza et al., 2013; Jacobs et al., 2013; Adhikari et al., 2019; Lona-Durazo et al., 2019). This result is also in line with what was reported in the CANDELA study (Adhikari et al., 2019), in which, compared with hair color, they identified a single candidate SNP in the *TYR* locus associated with eye color. This exemplifies the importance of characterizing the genetic architecture of different pigmentation traits independently and opens up new questions to investigate the different mechanisms involved in melanin synthesis between cutaneous versus iris melanocytes.

We conducted TWAS and colocalization analysis with expression and methylation QTLs to further explore the shared causal signals among these phenotypes. Through colocalization with melanocyte eQTLs, we found colocalization in the *OCA2* region, likely regulated by the SNP rs12913832 in the nearby *HERC2* gene. Similarly, colocalization with meQTLs highlighted a signal in the *HERC2* locus. The shared signals between meQTLs, eQTLS, and eye color GWAS hits in the *HERC2* region may suggest that DNA methylation could play a role in the differential expression of *OCA2*, thus influencing the eye color phenotype, although this cannot be confirmed with the current evidence. Further analyses, such as Mendelian randomization, will be useful to evaluate causal associations among these traits (e.g. Bonilla et al., 2020).

We did not find colocalization of GWAS SNPs with eQTLs on the *TYRP1* locus, even though our GWAS and fine-mapping results suggest a regulatory role of the candidate causal variants in this locus due to (1) the location ∼11kb upstream of the gene and (2) the overlap of a SNP (rs13297008) with open chromatin regions in foreskin melanocytes. In addition, this gene was absent from the TWAS expression weights dataset, suggesting that the gene expression in the current dataset is not sufficiently heritable (i.e. heritability p > 0.01). Therefore, we are not able to provide evidence of the mechanism in which the variants in the locus affect pigmentation variation, nor we are able to nominate a single causal SNP.

The cultured melanocyte expression and methylation QTLs we used for colocalization and TWAS come from newborn foreskin melanocytes (Zhang et al., 2018, 2021). Similarly, the regulatory annotations from the ENCODE and Roadmap Epigenomics projects (Dunham et al., 2012; Roadmap Epigenomics Consortium et al., 2015) also come from melanocytes, keratinocytes, and fibroblasts from foreskin tissue. The melanocytes from skin and iris have several similarities and same embryological origin, but there are also significant differences between them. For instance, the melanosomes within the iris melanocytes are retained in the cytoplasm, and they are not transferred through dendrite-like structures to adjacent keratinocytes, as it is the case in the skin and hair melanocytes (Sturm and Frudakis, 2004). Moreover, the iris melanocytes are not reactive to the alpha melanocyte stimulating hormone (α-MSH) (Li et al., 2006), and instead, alternative signaling cascades trigger and regulate melanogenesis (Zhou et al., 2018). Therefore, future QTL efforts using a more precise tissue type, such as uveal melanocytes, may aid in characterizing the regulatory differences between cutaneous and iris melanocytes.

The *HERC2/OCA2* region on chromosome 15 has the strongest effect on eye color variation, such that blue eye color was initially considered a Mendelian trait (Davenport and Davenport, 1907; Sturm and Frudakis, 2004). The most significant variant associated with blue versus brown eye color is rs12913832, an enhancer of the expression of *OCA2* (Visser et al., 2012), whereas the same polymorphism only causes a mild decrease of hair and skin eumelanin content, suggesting that the effect of this locus is different between dermal and iris melanocytes (Visser et al., 2014). Our findings suggest that it is likely that other SNPs in the locus also have an effect on the expression of *OCA2* in the iris. For instance, a subset of participants harbored the rs12913832 homozygous genotype associated with blue eye color (i.e. GG), but they self-reported non-blue eye color. In addition, there may be rare genetic variants within the OCA2/HERC2 region (i.e. rs121918166 and rs74653330) accounting for the blue eye color individuals under the heterozygous rs12913832 genotype, but larger sample size studies are needed to statistically test this hypothesis. Therefore, the expression of *OCA2* might be induced by other regulatory variants in the locus, counteracting the effect of rs12913832, as has been previously proposed (Andersen et al., 2016). An alternative explanation could be that a subset of participants self-reported their eye color inaccurately, a hypothesis that we are not able to discard.

In addition, it is possible that genetic interactions between *IRF4* and *OCA2* also play a role. For instance, it is known that individuals may have blue eye color when harboring one or two rs12913832 A-alleles (*HERC2*), associated with nonblue eye color, along with one or two rs12203592 T-alleles, associated with light eye color (Laino et al., 2018). Similarly, in the CanPath there is a significant increase in the number of nonbrown eye color individuals with the rs129138-AG genotype as the number of rs12203592-T alleles increases (Fisher exact test p value = 2.2e-16) (See Tables S8 and S9 and Figure S9). Finally, it has been recently suggested that SNPs in the genes *TYR* (rs1126809)*, TYRP1* (rs35866166, rs62538956), and *SLC24A4* (rs1289469) may be responsible for the brown eye color in individuals of European ancestry with an rs12913832 homozygous G-allele background (Meyer et al., 2020). However, our formal interaction analyses using CASSI did not identify significant interactions (after Bonferroni correction) between any pair of markers analyzed, including the polymorphisms rs12913832 (*HERC2*) and rs12203592 (*IRF4*).

Genetic correlations among hair and eye color in the CanPath cohort are high, in line with what has been previously reported (Lin et al., 2016) and considering the effect that most genes have in both phenotypes too (e.g. *SLC24A4, IRF4, OCA2*). However, we have demonstrated that certain genetic differences come to light when investigating candidate causal variants across the genome. Within the CanPath cohort, we observed that red hair color is driven mainly by multiple candidate causal signals in the *MC1R* locus and that variants within the same gene also have a significant effect upon blonde hair color. In contrast, variants within *MC1R* and its antagonist, *ASIP*, are not associated with eye color, which may be explained by the fact that *MC1R* is not expressed in iris melanocytes (Li et al., 2006). In addition, this may explain why iris melanocytes do not respond to UV radiation as opposed to skin melanocytes. *HERC2/OCA2* is the most significant locus in our analysis of blond versus black and brown versus black hair color (although as described earlier, *MC1R* is the most important locus determining red hair color). *HERC2/OCA2* is also the most significant locus for eye color. However, the signal from hair color is primarily driven by rs12913832, whereas there are several independent signals in *HERC2/OCA2* associated with eye color. Lastly, even though *IRF4*, a transcription factor that upregulates tyrosinase, has a large effect on both blonde hair and blue eye color, the direction of effect of the causal SNP rs12203592 is opposite for both traits: the derived T-allele is associated with blue eye color, whereas the same allele is associated with the presence of brown hair color (Praetorius et al., 2013).

### Limitations of the study

The main caveat of the study is the self-reported eye color from CanPath participants.

## STAR★Methods

### Key resources table

**Table:** 

REAGENT or RESOURCE	SOURCE	IDENTIFIER
**Deposited data**		

GWAS summary statistics	This study	https://canpath.ca/
Melanocyte genotype data	(Zhang et al., 2018)	dbGaP phs001500.v1.p1
RNA-seq expression data	(Zhang et al., 2018)	dbGaP phs001500.v1.p1
meQTL association results	(Zhang et al., 2021)	dbGaP phs001500.v1.p1

**Software and algorithms**		

QC Perl script	McCarthy Group Tools	https://www.well.ox.ac.uk/∼wrayner/tools/
PLINK 1.9	PLINK Working Group (Purcell et al., 2007; Chang et al., 2015)	https://www.cog-genomics.org/plink/
Sanger Imputation Server	Wellcome Sanger Institute (McCarthy et al., 2016)	https://www.sanger.ac.uk/tool/sanger-imputation-service/
PLINK 2.0	PLINK Working Group (Purcell et al., 2007; Chang et al., 2015)	https://www.cog-genomics.org/plink/2.0/
R version 3.5.1	R Core Team (R Core Team, 2019)	https://www.r-project.org/
GCTA version 1.26.0	(Yang et al., 2011, 2012, 2014)	https://yanglab.westlake.edu.cn/software/gcta/#Overview
METASOFT version 2.0.1	(Han and Eskin, 2011)	http://genetics.cs.ucla.edu/meta/
LocusZoom	(Pruim et al., 2011)	http://locuszoom.org/
SNPNexus	(Ullah, Lemoine and Chelala, 2012; Ullah et al., 2018)	https://www.snp-nexus.org/v4/
FINEMAP version 1.4	(Benner et al., 2016)	http://www.christianbenner.com/
LDStore version 2.0	(Benner et al., 2016)	http://www.christianbenner.com/#
HaploReg version 4	(Ward and Kellis, 2012, 2016)	https://pubs.broadinstitute.org/mammals/haploreg/haploreg.php
CASSI	(Howey, 2017)	https://www.staff.ncl.ac.uk/richard.howey/cassi/using.html
HyPrColoc	(Foley et al., 2021)	https://github.com/jrs95/hyprcoloc
FUSION	(Gusev et al., 2016)	http://gusevlab.org/projects/fusion/

### Resource availability

#### Lead contact

Further information and requests for resources and reagents should be directed to and will be fulfilled by the lead contact: Frida Lona-Durazo (frida.lona-durazo@mail.utoronto.ca).

#### Materials availability

This study did not generate new unique reagents.

### Experimental model and subject details

This study was approved by the University of Toronto Ethics Committee (Human Research Protocol # 36429) and data access was granted by the Canadian Partnership for Tomorrow’s Health (Application number DAO-034431). The samples in this study correspond to a subset of 5,675 individuals from the Canadian Partnership for Tomorrow’s Health (CanPath), which were sampled in different provinces: Alberta (N = 926; 16.4%), Atlantic Coast Provinces (i.e. New Brunswick, Newfoundland, Nova Scotia and Prince Edward Island) (N = 385; 6.8%), British Columbia (N = 965; 17.1%), Ontario (N = 934; 16.5%) and Quebec (N = 2434; 43.1%). We selected the individuals who self-reported having European-related ancestry and for whom self-reported eye colour was available (N = 5,641), of which 58.78% were females. The average age across participants was 55 years old (SE ± 0.11).

### Method details

#### Genotyping of participants and quality control

Individuals who self-reported as having European-related ancestry were genotyped between 2012 and 2018 using two different genotyping array chips: Axiom 2.0 UK Biobank (Affymetrix) (N = 3,212) and the Global Screening Array (GSA) 24v1+MDP (N = 2,429) by the Canadian Partnership for Tomorrow’s Health (CanPath). The number of single nucleotide polymorphisms (SNPs) of these chip arrays ranges between 658,296 and 813,168 SNPs.

We performed genotype quality control for each array chip separately by first filtering out variants that deviated in minor allele frequency >0.2 from the 1000 Genomes Project Phase 3 European sample (1KGP-EUR), GC/TA variants with minor allele frequency >0.4 in the 1KGP-EUR and flipping alleles according to the 1KGP-EUR, using a Perl script (version 4.2) (Rayner, 2019). Afterwards, we used PLINK (version 1.9) (Purcell et al., 2007; Chang et al., 2015) to filter out variants with minor allele frequency <1%, high missing genotyping rate (--geno 0.05), high missing individual rate (--mind 0.05) or variants that significantly deviated from the Hardy-Weinberg Equilibrium (--hwe 1e-06). Then, we also identified second-degree relatives (--genome, PI_HAT >0.2) using a pruned set of variants in linkage disequilibrium (LD) (--indep-pairwise 100 10 0.1), and filtered out, from each pair, the individual with the lowest genotyping rate. Finally, we performed a Principal Components Analysis (PCA) of a pruned set of common variants of our study samples projected on the 1KGP Phase 3 samples on PLINK (version 1.9) (Purcell et al., 2007; Chang et al., 2015), and removed individual outliers that did not cluster within the European sample of the 1KGP by inspecting the first three principal components (total PCA outliers across genotyping arrays = 81). Amongst the outliers, 63 individuals are from Quebec, 8 from British Columbia, 5 from the Atlantic Provinces, 5 from Alberta and none from Ontario.

#### Imputation of genotypes

Each genotyping array was first phased with EAGLE2 (version 2.0.5) (Loh et al., 2016) using the Sanger Imputation Server (McCarthy et al., 2016). After phasing, samples on each genotyping array were imputed on the Sanger Imputation Server using the positional Burrows-Wheeler transform (PBWT) algorithm (Durbin, 2014) and the Haplotype Reference Consortium (HRC) release 1.1 dataset as reference (McCarthy et al., 2016). The HRC includes ∼64,000 haplotypes and ∼40,000,000 autosomal SNPs of ∼32,000 individuals predominantly of European ancestry, which makes it ideal for the imputation of our datasets, which are of European-related ancestry. After imputation, we used PLINK (version 2) (Purcell et al., 2007; Chang et al., 2015) to filter out variants with minor allele frequency <1%, high missing genotyping rate (--geno 0.05), imputation score (INFO) < 0.3, or variants that significantly deviated from the Hardy-Weinberg Equilibrium (--hwe 1e-06).

#### Phenotyping

Participants of the CanPath answered a questionnaire that included self-report on eye colour using the following discrete categories: grey, blue, green, amber, hazel or brown eye colour. These categories were then transformed into a linear scale using R (version 3.5.1) (R Core Team, 2019) to build a linear model with the following levels: 1 = grey or blue, 2 = green, 3 = hazel, 4 = brown. Table S2 shows the number of individuals on each eye colour category by genotyping array. In addition, participants also reported their age and sex. We excluded the individuals who reported amber eye colour, due to the low sample count.

#### Genome-wide association studies (GWAS) and meta-analyses

Genome-wide association studies of eye colour were performed for each genotyping array with a linear mixed model on Genome-Wide Complex Trait Analysis (GCTA- MLMA) 1.26.0 (Yang et al., 2011, 2014), using an additive genetic model (i.e. the effect size is a linear function of the number of effect alleles). We performed a PCA of a pruned set of genotyped variants for each genotyping array after quality control, keeping only SNPs with MAF >0.05 and excluding regions of high LD, using PLINK (version 1.9) (Purcell et al., 2007; Chang et al., 2015). We included in the model sex, age and the first ten PCs as fixed effects, and a genetic relationship matrix (GRM) of genotyped SNPs computed on GCTA 1.26.0 (Yang et al., 2011, 2014) as random effects, to control for more subtle population structure. To evaluate the case of residual population substructure, we computed the expected vs. observed p-values using Q-Q plots on R (version 3.5.1) (R Core Team, 2019), and ran LD Score regression with LDSC, in which an LD Score intercept considerably higher than 1 may indicate remaining confounding bias (Bulik-Sullivan et al., 2015).

We performed a meta-analysis of eye colour using the beta coefficient and standard error (SE) of each study on the software METASOFT (version 2.0.1) (Han and Eskin, 2011). METASOFT conducts a meta-analysis using a fixed effects model (FE), which works well when there is no evidence of heterogeneity (i.e. assumes same effect size across studies), and an optimized random effects model (RE2), which works well when there is evidence of heterogeneity among studies (Han and Eskin, 2011). Additionally, METASOFT computes two estimates of statistical heterogeneity, Cochran’s Q statistic and I^2^, as well as a Bayesian posterior probability that an effect exists on each individual study (M) (Han and Eskin, 2012).

For the meta-analyses results, we generated Manhattan and Q-Q plots using the qqman (Turner, 2018) and ggplot2 (Wickham et al., 2019) R packages. In addition, we visualized the significant loci with regional plots using the web-based program LocusZoom (Pruim et al., 2011), with the 1KGP Phase 3 European sample as reference LD. We focused our results on the fixed effects model, but we also report the RE2 on the summary statistics of the top signals as Data S1, and compared the statistical significance between both models when there was evidence of heterogeneity based on Cochran’s Q p-value and I^2^ statistics.

#### Annotation of significant loci

We used the web-based program SNPNexus (Ullah et al., 2012; Ullah et al., 2018) to annotate the genome-wide significant signals (p value < 1e-08) from the meta-analysis. Specifically, gene and variant type annotation were done using the University of California Santa Cruz (UCSC) and Ensembl databases (human genome version hg19); assessment of the predictive effect of non-synonymous coding variants on protein function was done with SIFT and PolyPhen scores. Both SIFT and PolyPhen output qualitative prediction scores (i.e. probably damaging/deleterious, possibly damaging/deleterious-low confidence, tolerated/benign). Non-coding variation scoring was assessed using CADD score, which is based on ranking the deleteriousness of a variant relative to all possible substitutions of the human genome. For instance, a score ≥ 20 indicates that the variant is predicted to be in the top 1% most deleterious variants in the genome (Ullah et al., 2018). In addition, we explored the effect of significant loci on RNA and protein expression using the GTEx database (Consortium et al., 2017) and the effect of significant genes using the Protein Atlas (Uhlén et al., 2015).

#### Approximate conditional analyses of association

In order to identify if the genome-wide significant loci of our original logistic meta-analyses were driven by one or more independent variants, we conducted approximate conditional and joint analyses of association (COJO) with GCTA (Yang et al., 2012). We performed the analysis (--cojo-slct) using as input the summary statistics of our eye colour meta-analysis (fixed effects, FE) and the weighted average effect allele frequency from all studies. In addition, the program requires a reference sample for computing LD correlations and, in the case of a meta-analysis, it is suggested to use one of the study’s large samples (Yang et al., 2012). Therefore, we ran the analysis twice: 1) using as a reference the sample genotyped with the Axiom UKBB array, and 2) using as a reference the sample genotyped with the GSA 24v1+MDP. We assumed that variants farther than 10 Mb are in complete linkage equilibrium and used a p-value threshold of 5e-08.

#### Statistical fine-mapping of significant loci

We used the program FINEMAP (version 1.4) (Benner et al., 2016) to identify candidate causal variants in the genome-wide associated loci across the genome for eye colour. FINEMAP is based on a Bayesian framework, which uses summary statistics and LD correlations among variants to compute the posterior probabilities of causal variants, with a shotgun stochastic search algorithm (Benner et al., 2016). Compared to other methods, FINEMAP allows a maximum of 20 causal variants per locus. To run the program, we used as input the meta-analysis summary statistics, including the weighted average MAF across all studies, and an LD correlation matrix from one of the large samples in our study (Axiom UKBB array, N = 4,745). The LD correlation matrix was computed using LDStore (version 2.0), which considers genotype probabilities (Benner et al., 2017). We defined regions for fine-mapping as ± 500 kb regions flanking the lead SNP, based on the genome-wide and suggestive signals of association from the meta-analyses, and setting the maximum number of causal SNPs to 10 for each locus (i.e. a maximum of 10 credible sets). A credible set is comprised of SNPs that cumulatively reach a probability of at least 95%. The SNPs within a credible set are referred to as candidate causal variants and each of them has a corresponding posterior inclusion probability (PIP).

We filtered FINEMAP results by removing candidate causal variants with a log_10_BF < 2 from each of the 95% credible sets, where a log_10_BF indicates considerable evidence of causality. We annotated the remaining SNPs using SNPnexus (Ullah et al., 2018) to obtain information about the overlapping/nearest genes, overlapping regulatory elements and CADD scores. Annotation of gene expression on ENCODE, Roadmap Epigenomics and Ensembl Regulatory Build was restricted to melanocytes and fibroblasts, which are the relevant cell types involved in eye colour. Based on the combined evidence of fine-mapping and posterior annotation, we defined the candidate causal variants with strong evidence of causality (based on their log_10_BF and annotation) as the most likely candidate causal variants. We computed LD correlations between the candidate causal SNP(s) on each locus (i.e. configuration with highest posterior probability and *k* number of SNPs) and the other variants on each locus using LDStore (version 2.0) and plotted the Posterior Inclusion Probability (PIP) results on R (version 3.5.1) (R Core Team, 2019) using ggplot2 (Wickham et al., 2019).

Given that there may be candidate functional SNPs that we did not genotype or did not impute with high accuracy, we explored if markers in the same LD blocks of the credible sets have functional annotations using HaploReg (version 4) (Ward and Kellis, 2012, 2016). We used as input the most likely candidate causal SNP on each credible set, the LD from the 1KGP European population with a threshold of r^2^ ≥ 0.8 and the core chromatin 15-state model, which is based on several histone marks associated with promoters, enhancers, insulators and heterochromatin.

#### SNP-SNP interactions

We computed statistical interactions using the program CASSI (Howey, 2017) across the index GWAS SNPs that reached a genome-wide significance threshold (*IRF4*: rs12203592, *TYRP1*: rs1326779; *TYR*: rs1126809; *SLC24A4*: rs4144266), and including as well the five loci fine mapped in the *OCA2/HERC2* region (rs4778138, rs12913832, rs117007668, rs117744568, rs71467328) using a linear regression model and the same covariates as in the GWAS. We used a Bonferroni-corrected p-value threshold to account for all the tests performed (0.05/33 tests = 0.0015).

#### Colocalization with expression and methylation QTLs from cultured melanocytes

We conducted colocalization analyses of our GWAS meta-analyses results with gene expression and methylation *cis-*QTL data from primary cultures of foreskin melanocytes, isolated from foreskin of 106 newborn males (Zhang et al., 2018, 2021). *Cis*-QTLs were assessed for variants in the ± 1Mb region of each gene or CpG. We used the program hyprcoloc (Foley et al., 2021) to obtain the posterior probability of a variant being shared between the eye colour GWAS signals and the expression or methylation QTLs. We tested all the significant eQTL genes or meQTL probes within ± 250 kb regions flanking the most significant GWAS SNP on each of the genome-wide regions (p-value ≤ 5e-8) from the meta-analysis summary statistics (five different loci). We used as LD reference the matrix obtained from the CanPath’s Axiom UKBB Array (INFO score >0.3), computed on PLINK (version 1.9; --r square) (Purcell et al., 2007; Chang et al., 2015). We kept colocalized regions that reached a posterior probability ≥0.8, indicating high confidence of shared causality.

#### Transcriptome-wide association studies

We performed a transcriptome-wide association study (TWAS) by imputing the expression profile of the CanPath cohort using GWAS summary statistics and melanocyte RNA-seq expression data (Zhang et al., 2018). Using the program FUSION (Gusev et al., 2016), we used as LD reference the CanPath’s Axiom UKBB genotyping array computed in binary PLINK format (version 1.9; --make-bed) (Purcell et al., 2007; Chang et al., 2015). As recommended by FUSION, we used the LDSC *munge_sumstats.py* script to check the GWAS summary statistics (90). Before running the script, we filtered out SNPs with MAF <0.01, SNPs with a genotyping missing rate >0.01 and SNPs that failed Hardy-Weinberg test at significance threshold of 1 × 10^−7^ using PLINK (version 1.9; --maf 0.01, --geno 0.01, --hwe 10e-7) (Purcell et al., 2007; Chang et al., 2015). We computed functional weights from our melanocyte RNA-seq data one gene at a time. Genes that failed quality control during a heritability check (using minimum heritability p-value of 0.01) were excluded from the further analyses, yielding a total of 3998 genes. We restricted the locus to 500 kb on either side of the gene boundary. We applied a significance cut-off to the final TWAS result of 1.25e-5 (i.e. 0.05/3998 genes tested). Finally, we performed conditional analysis on FUSION (FUSION_post.process.R script) if more than one gene in a locus was significant, to identify if these were independent signals.

#### Genetic correlations

We used a bivariate restricted maximum likelihood (REML) approach to test for genome-wide pleiotropy between hair and eye colour using GCTA (--reml-bivar option) (Yang et al., 2012), by taking advantage of the hair colour meta-analysis dataset from CanPath (Lona-Durazo et al., 2021). To consider the whole spectrum of colour in both traits, and thus maximize the number of loci, we coded both traits on a linear scale (excluding red hair colour). Hair colour ranged from 1= blonde, 2=light brown, 3= dark brown, and 4= black, whereas eye colour categories ranged from 1= grey/blue, 2= green, 3= hazel, and 4= brown. Given that the program requires genotype-level data, we computed the analysis twice, using the two largest samples for which eye colour data was available: Axiom UKBB array (N= 3,212) and GSA 24v1+MDP (N=2,429). Significance of the genetic correlations was computed with a likelihood ratio test on R (version 3.5.1) (R Core Team, 2019).

We first included in the model sex, age and the significant PCs as covariates, and restricted the analysis to SNPs with high INFO score (i.e. INFO >0.8) and MAF >1%. We then explored the correlations between each phenotype (i.e. hair and eye colour) and the eigenvectors of the principal components analysis. If the eigenvectors are correlated with the ancestry (i.e. geography) of the individuals, setting them as covariates may hinder the true genetic correlation between both traits, given that hair and eye colour are themselves correlated with ancestry. Therefore, we ran the genetic correlation a second time using as covariates only the non-significant principal components. In the case of the Axiom UKBB array we used PC3 and PC5, and in the case of GSA 24v1+MDP we used PC4, PC5 and PC8.

### Quantification and statistical analysis

The quantitative and statistical analyses are described in the relevant sections of the Method details or in the table and figure legends.

## Data Availability

The datasets supporting this manuscript are included as supplemental information. We provide the genome-wide (p ≤ 5e-8) and suggestive (p ≤ 1e-6) signals identified in the eye colour GWAS meta-analysis as a supplemental information File (Data S1). Further information and requests for data published here should be directed to CanPath, which regulates the access to the data and biological materials (https://canpath.ca/). Melanocyte genotype data, RNA-seq expression data, and all meQTL association results have been deposited in Genotypes and Phenotypes (dbGaP) under accession dbGaP: phs001500.v1.p1. This paper does not report original code. Any additional information required to reanalyze the data reported in this paper is available from the lead contact upon request.

## Supplemental information

Document S1. Figures S1–S9 and Tables S1–S9

Data S1. Genome-wide (p ≤ 5e-8) and suggestive (p ≤ 1e-6) signals identified in the eye colour GWAS meta-analysis, related to Figure 2

Data S2. FINEMAP results and annotation of loci with logBF ≥ 2, related to STAR Methods
