# Supplementary material for: Investigating the genetic architecture of eye colour in a Canadian cohort
Source: iScience. 2022 May 30;25(6):104485. doi: 10.1016/j.isci.2022.104485 (PMC9194134; doi:10.1016/j.isci.2022.104485)
Supplement: Document S1. Figures S1–S9 and Tables S1–S9 [file mmc1.pdf]

iScience, Volume *25*

Supplemental information

Investigating the genetic architecture of eye colour in a Canadian cohort

Frida Lona-Durazo, Rohit Thakur, Erola Pairo-Castineira, Karen Funderburk, Tongwu Zhang, Michael A. Kovacs, Jiyeon Choi, Ian J. Jackson, Kevin M. Brown, and Esteban J. Parra

Supplementary Tables

Table S1. Allele frequency across provinces in the CanPath for the rs12913832 *HERC2* variant, Related STAR Methods. QC = Quebec; ATL = Atlantic Provinces; ATP = Alberta; BC = British Columbia; ON = Ontario; all-noQC = all provinces, excluding Quebec.

	A_allele	G_allele
**QC**	0.373	0.627
**ATL**	0.232	0.768
**ATP**	0.227	0.773
**BC**	0.237	0.763
**ON**	0.242	0.758
**all-noQC**	0.235	0.765

Table S2. Number of individuals included on each eye colour category, by genotyping array and combined in the meta-analyses, Related to STAR Methods.

Eye Colour	Axiom UKBB	Illumina GSA	Meta-Analysis
**Blue + grey**	1,371	758	2,129
**Green**	481	569	1,050
**Hazel**	665	547	1,212
**Brown**	695	555	1,250
**Total**	3,212	2,429	5,641

Table S3. Conditional and Joint Analysis of Association (GCTA-COJO) results of eye colour, in which LD was calculated using the Axiom UKBB genotyping array, Related to STAR Methods. Position= chromosomal position (hg19); AF= allele frequency; SE= standard error; FE= fixed effects model; RE2= random effects model; LD: linkage disequilibrium with the SNP in the line below; I^2= heterogeneity measure, where I^2 > 50 is considered as notable heterogeneity.

chromosome	rsid	position	Ref Allele	Ref AF	beta	SE	p-value (FE)	p-value (RE2)	beta_joint	se_joint	p-value_joint	Nearest Gene	LD (r^2)	I^2	Cochran's Q	Cochran's Q p-value
6	rs12203592	396321	T	0.17	-0.16	0.03	8.80E-09	1.24E-08	-0.16	0.03	9.67E-09	*IRF4*	0.0	23.04	1.30	0.25
6	rs116072038	34650865	G	0.02	-0.44	0.08	1.46E-08	2.07E-08	-0.44	0.08	1.62E-08	*ILRUN*	0.0	0.00	0.71	0.40
9	rs1326779	12737581	C	0.30	0.14	0.02	4.74E-09	6.79E-09	0.14	0.02	5.26E-09	*TYRP1*	0.0	52.20	2.09	0.15
11	rs1126809	89017961	A	0.28	-0.14	0.02	3.13E-08	3.96E-08	-0.14	0.02	3.41E-08	*TYR*	0.0	0.00	0.04	0.85
14	rs4144266	92789205	G	0.44	-0.12	0.02	1.94E-08	2.34E-08	-0.12	0.02	2.12E-08	*SLC24A4*	0.0	69.55	3.28	0.07
15	rs4778138	28335820	G	0.13	0.81	0.03	2.89E-164	1.71E-163	0.32	0.04	1.16E-16	*OCA2*	0.2	0.00	0.30	0.58
15	rs1129038	28356859	T	0.76	-1.24	0.02	0	0	-0.76	0.04	1.98E-81	*HERC2*	0.1	95.14	20.56	5.77E-06
15	rs79380392	28409219	T	0.02	1.11	0.08	8.37E-49	6.57E-49	0.62	0.08	8.97E-14	*HERC2*	0.0	86.70	7.52	0.01
15	rs72714147	28478799	A	0.08	0.84	0.04	4.41E-110	3.39E-112	0.52	0.05	7.98E-31	*HERC2*	0.0	94.86	19.45	1.03E-05
15	rs117744568	28498692	A	0.02	0.87	0.06	1.09E-45	2.70E-45	0.73	0.06	2.31E-30	*HERC2*	0.0	74.88	3.98	0.05
15	rs10162958	28507825	G	0.05	0.95	0.04	4.45E-105	2.15E-105	0.49	0.05	2.03E-20	*HERC2*	0.0	89.78	9.78	1.76E-03

Table S4. Conditional and Joint Analysis of Association (GCTA-COJO) of eye colour, in which LD was calculated using the Illumina GSA array, Related to STAR Methods. Position= chromosomal position (hg19); AF= allele frequency; SE= standard error; FE= fixed effects model; LD: linkage disequilibrium with the SNP in the line below. Related to Results.

chromosome	rsid	position	Ref Allele	Ref AF	beta	SE	p-value (FE)	beta_joint	se_joint	p-value_joint	Nearest Gene	LD (r^2)
6	rs12203592	396321	T	0.17	-0.17	0.03	8.80E-09	-0.17	0.03	9.67E-09	*IRF4*	0
6	rs116072038	34650865	G	0.02	-0.44	0.08	1.33E-08	-0.44	0.08	1.46E-08	*ILRUN*	0
9	rs1326779	12737581	C	0.3	0.14	0.02	4.74E-09	0.14	0.02	5.26E-09	*TYRP1*	0
11	rs1126809	89017961	A	0.28	-0.14	0.03	3.13E-08	-0.14	0.03	3.41E-08	*TYR*	0
14	rs4144266	92789205	G	0.44	-0.12	0.02	1.94E-08	-0.12	0.02	2.12E-08	*SLC24A4*	0
15	rs4778239	28337939	T	0.99	-0.94	0.1	2.72E-21	-0.78	0.1	1.59E-14	*OCA2*	0.02
15	rs1129038	28356859	T	0.76	-1.24	0.02	0	-0.69	0.04	1.17E-70	*HERC2*	0.03
15	rs116879078	28363488	T	0.01	0.88	0.1	3.73E-18	0.84	0.1	8.22E-16	*HERC2*	0
15	rs79380392	28409219	T	0.02	1.11	0.08	8.37E-49	1.01	0.08	1.89E-36	*HERC2*	0
15	rs72714147	28478799	A	0.08	0.84	0.04	4.41E-110	0.67	0.05	5.39E-51	*HERC2*	0.01
15	rs8028689	28488888	C	0.06	0.96	0.04	1.71E-118	0.81	0.05	8.77E-64	*HERC2*	0
15	rs117744568	28498692	A	0.02	0.87	0.06	1.09E-45	0.82	0.07	2.60E-36	*HERC2*	0

Table S5. Nominally significant SNPs replicated from the independent signals identified by Simcoe *et al.* 2021, Related to Results.

Simcoe et al. 2021									CanPath (this study)									Concordance
chromosome	position	rsid	Nearest Gene	Ref Allele	Ref AF	Beta	SE	p-value	EA	NEA	beta (FE)	SE	p-value	I^2	Cochran's Q	Cochran's Q p-value		
1	212421629	rs351385	*DTL*	G	0.580	-0.083	0.008	2.31E-28	G	A	-0.049	0.022	0.027	35.154	1.542	0.214		Yes
2	219755011	rs121908120	*WNT10A*	T	0.976	0.140	0.025	1.54E-08	A	T	-0.178	0.079	0.025	0.000	0.732	0.392		Yes
2	239276278	rs74409360	*TRAF3IP1*	T	0.076	-0.128	0.014	8.09E-20	T	C	-0.096	0.040	0.017	0.000	0.695	0.404		Yes
3	69980177	rs116359091	*MITF*	G	0.974	-0.229	0.025	1.02E-20	A	G	0.231	0.073	0.002	0.000	0.424	0.515		Yes
5	311902	rs62330021	*PDCD6,AHRR*	G	0.948	-0.325	0.017	1.36E-80	A	G	0.117	0.053	0.026	0.000	0.007	0.931		Yes
5	148206885	rs1800888	*ADRB2*	T	0.013	-0.229	0.033	5.20E-12	T	C	-0.240	0.082	0.003	0.000	0.073	0.786		Yes
6	396321	rs12203592	*IRF4*	T	0.171	-0.385	0.010	1.61E-321	T	C	-0.166	0.029	6.39E-09	13.740	1.159	0.282		Yes
6	10538183	rs6910861	*GCNT2*	G	0.461	0.078	0.008	1.20E-24	G	A	0.056	0.022	0.010	0.000	0.000	0.997		Yes
9	12677471	rs13297008	*TYRP1*	G	0.388	0.260	0.008	4.99E-250	A	G	-0.128	0.022	8.76E-09	0.000	0.698	0.404		Yes
9	12679244	rs62538956	*TYRP1*	T	0.882	-0.282	0.012	5.43E-130	C	T	0.147	0.035	2.04E-05	0.000	0.021	0.886		Yes
11	88902144	rs10830237	*TYR*	T	0.590	0.102	0.008	3.27E-41	T	C	0.044	0.022	0.045	0.000	0.231	0.631		Yes
11	89017961	rs1126809	*TYR*	G	0.721	0.285	0.008	1.82E-255	A	G	-0.136	0.025	3.17E-08	0.000	0.016	0.900		Yes
15	27900576	rs34422827	*OCA2*	T	0.468	-0.138	0.008	1.29E-75	G	T	0.070	0.022	0.001	0.000	0.017	0.896		Yes
15	28198772	rs117616283	*OCA2*	G	0.976	-1.639	0.033	<1E-330	A	G	0.357	0.063	1.21E-08	58.909	2.434	0.119		Yes
15	28241020	rs4778224	*OCA2*	G	0.786	-0.282	0.010	8.18E-162	G	A	-0.159	0.029	5.13E-08	75.296	4.048	0.044		Yes
15	28288121	rs1470608	*OCA2*	T	0.151	0.962	0.010	<1E-330	T	G	0.495	0.030	1.89E-59	70.954	3.443	0.064		Yes
15	28329346	rs151090307	*OCA2*	T	0.022	-0.895	0.033	6.20E-164	T	C	-0.605	0.128	2.20E-06	0.000	0.120	0.729		Yes
15	28330373	rs77542847	*OCA2*	C	0.922	-1.594	0.014	<1E-330	A	C	0.726	0.039	1.86E-77	94.077	16.882	0.000		Yes
15	28333189	rs76415938	*OCA2*	T	0.034	1.589	0.021	<1E-330	T	C	0.715	0.054	7.62E-40	0.000	0.318	0.573		Yes
15	28337939	rs4778239	*OCA2*	T	0.985	-2.115	0.034	<1E-330	T	C	-0.939	0.100	4.14E-21	76.997	4.347	0.037		Yes
15	28342069	rs8026089	*OCA2*	C	0.967	-1.571	0.021	<1E-330	A	C	0.935	0.068	1.04E-42	87.119	7.764	0.005		Yes
15	28356859	rs1129038	*HERC2*	T	0.734	-2.605	0.006	<1E-330	T	C	-1.233	0.024	0	95.244	21.026	0.000		Yes
15	28358104	rs77854840	*HERC2*	G	0.976	-2.256	0.026	<1E-330	A	G	1.043	0.070	1.02E-50	86.267	7.282	0.007		Yes
15	28384534	rs150287021	*HERC2*	T	0.012	2.626	0.041	<1E-330	T	C	0.949	0.158	2.01E-09	67.386	3.066	0.080		Yes
15	28530182	rs1667394	*HERC2*	T	0.822	-2.244	0.008	<1E-330	T	C	-1.037	0.027	0	95.081	20.329	0.000		Yes
21	44783957	rs619484	*SIK1*	G	0.634	-0.113	0.008	4.18E-48	A	G	0.051	0.023	0.023	70.468	3.386	0.066		Yes

Table S6. TWAS results showing the genome-wide significant genes in the TWAS (p-value ≤ 1.25e-5), Related to STAR Methods. GWAS/eQTL best SNP is the most significant SNP on each analysis. * = signals are not independent from each other, as evidenced by conditional TWAS.

Gene	Chromosome	GWAS best SNP	GWAS Z-score	eQTL best SNP	eQTL Z-score	# of SNPs	# weighted SNPs	TWAS Z-score	TWAS p-value
RIN3*	14	rs12896399	-5.56	rs12893289	-6.14	986	4	5.189	2.12E-07
SLC24A4*	14	rs12896399	-5.56	rs61977801	-6.68	933	11	5.287	1.24E-07
OCA2	15	rs12913832	53.28	rs12913832	6.24	489	1	53.279	0

Table S7. Statistical interactions results across all the top GWAS SNPs that reached a genome-wide significance threshold (*IRF4*: rs12203592, *TYRP1*: rs1326779; *TYR*: rs1126809; *SLC24A4*: rs4144266), and including also the five loci fine mapped in the *OCA2/HERC2* region (rs4778138, rs12913832, rs117007668, rs117744568, rs71467328), Related to STAR Methods.

SNP1	SNP2	beta	SE	pvalue
rs12203592	rs1326779	-0.161	0.137	0.241
rs12203592	rs1126809	-0.039	0.139	0.777
rs12203592	rs4144266	-0.131	0.129	0.310
rs12203592	rs4778138	-0.074	0.176	0.674
rs12203592	rs12913832	-0.013	0.139	0.925
rs12203592	rs117007668	-0.101	0.394	0.798
rs12203592	rs71467328	0.603	0.322	0.061
rs1326779	rs1126809	-0.024	0.116	0.835
rs1326779	rs4144266	0.068	0.106	0.524
rs1326779	rs117007668	0.125	0.324	0.700
rs1326779	rs117744568	-0.055	0.295	0.853
rs1326779	rs71467328	0.253	0.259	0.329
rs1126809	rs4144266	-0.113	0.111	0.306
rs1126809	rs4778138	-0.031	0.151	0.838
rs1126809	rs12913832	0.212	0.118	0.072
rs1126809	rs117007668	0.407	0.334	0.223
rs1126809	rs117744568	0.424	0.310	0.172
rs1126809	rs71467328	0.338	0.289	0.243
rs4144266	rs4778138	0.265	0.134	0.047
rs4144266	rs12913832	0.261	0.106	0.014
rs4144266	rs117007668	0.467	0.295	0.113
rs4144266	rs117744568	0.412	0.272	0.129
rs4144266	rs71467328	-0.052	0.243	0.831
rs4778138	rs12913832	-0.204	0.141	0.148
rs4778138	rs117007668	0.304	0.508	0.550
rs4778138	rs117744568	0.608	0.476	0.202
rs4778138	rs71467328	-0.401	0.336	0.232
rs12913832	rs117007668	-0.701	0.428	0.102
rs12913832	rs117744568	-0.617	0.349	0.078
rs12913832	rs71467328	-0.002	0.376	0.995
rs117007668	rs117744568	0.140	0.761	0.854
rs117007668	rs71467328	-1.878	1.084	0.083
rs117744568	rs71467328	-1.380	0.981	0.160

Table S8. Contingency table of the distribution of observed and expected brown vs. non-brown eye colour individuals with respect to the *IRF4* rs12203592 and *HERC2* rs12913832 genotypes, Related to STAR Methods. P-values were calculated using a Fisher's exact test.

	brown		non-brown		
HERC2:IRF4	observed	expected	observed	expected	p-value
**AA:CC**	255	243	134	146	
**AA:TC**	81	89	62	54	0.019
**AA:TT**	5	9	9	5	
**AG:CC**	693	621	846	918	
**AG:TC**	191	233	387	345	2.20E-16
**AG:TT**	4	33	78	49	
**GG:CC**	15	15	2006	2006	
**GG:TC**	5	6	769	768	0.749
**GG:TT**	1	1	100	100	

Table S9. Contingency table of the distribution of observed and expected blue vs. non-blue eye colour individuals with respect to the *IRF4* rs12203592 and *HERC2* rs12913832 genotypes, Related to STAR Methods. P-values were calculated using a Fisher's exact test.

	blue		non-blue		**p-value**
HERC2:IRF4	observed	expected	observed	expected	p-value
**AA:CC**	4	4	385	385	
**AA:TC**	2	2	141	141	0.711
**AA:TT**	0	0	14	14	
**AG:CC**	71	92	1468	1447	
**AG:TC**	42	34	536	544	1.22E-07
**AG:TT**	18	5	64	77	
**GG:CC**	1361	1390	660	631	
**GG:TC**	554	532	220	242	0.025
**GG:TT**	77	69	24	32	

Supplementary Figures

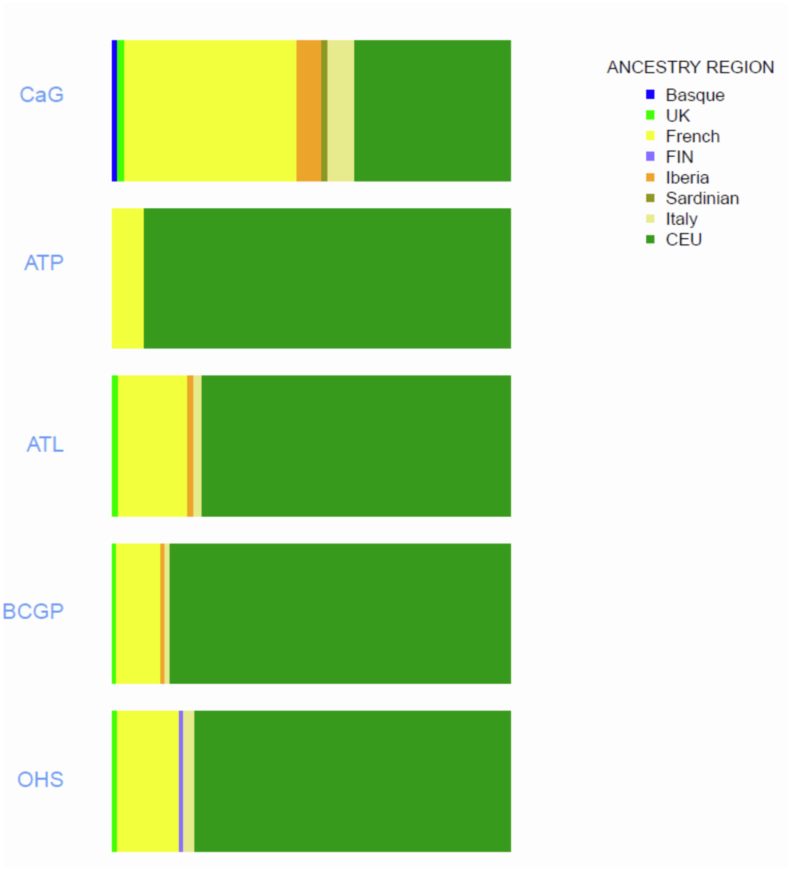

Figure S1. Ancestry proportions estimated from Chromopainter for each Canadian province, using as source the populations from the 1000 Genomes Project Phase 3 and Human Genome Diversity Project, Related to STAR Methods. ATL = Atlantic Provinces; ATP = Alberta; BCGP= British Columbia; CaG= Quebec, OHS= Ontario.

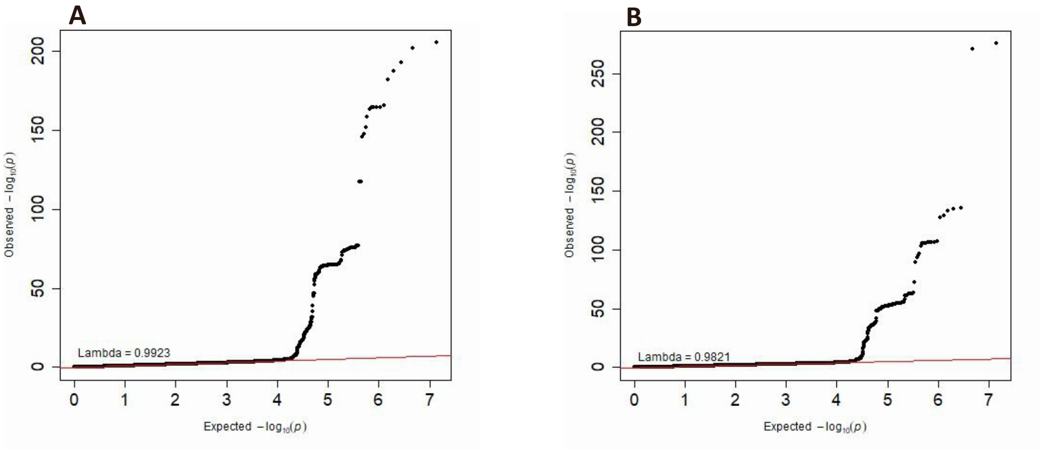

Figure S2. Q-Q plots (-$\log_{10}$ p-values) of each cohort for the GWAS showing the genomic inflation factor (lambda λ), Related to STAR Methods. **A.** Axiom UKBB array (λ = 0.9923). **B.** GSA Array (lambda = 0.9821).

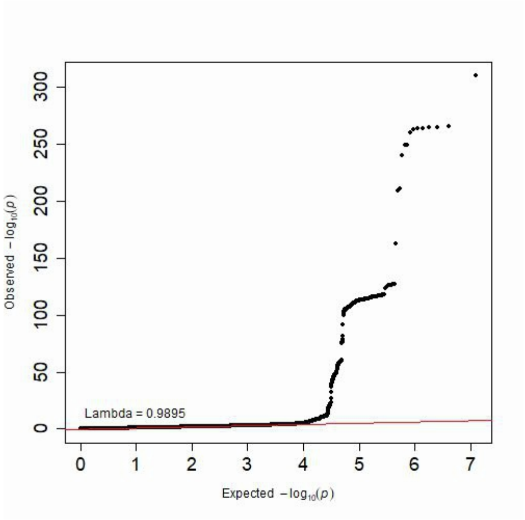

Figure S3. Q-Q plot (-$\log_{10}$ p-values) of meta-analysis showing the genomic inflation factor (λ = 0.9895), Related to Figure 2.

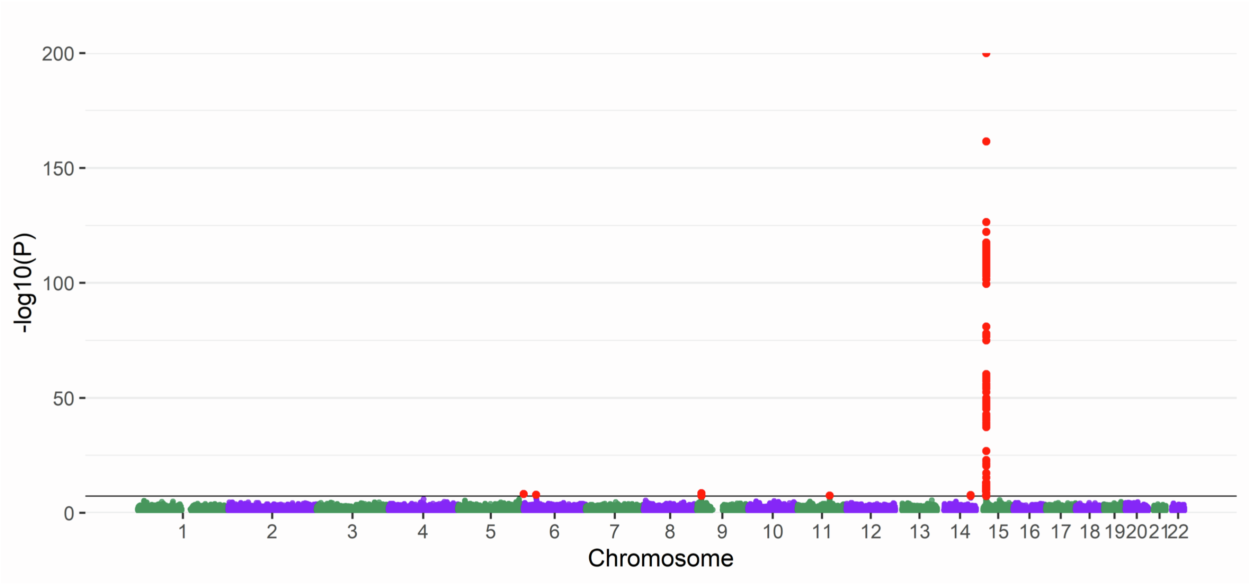

Figure S4. Manhattan plot of eye colour meta-analysis based on a linear mixed model, Related to Figure 2. The continuous line denotes the genome-wide threshold (p= 5e-8). Markers in red are genome-wide significant.

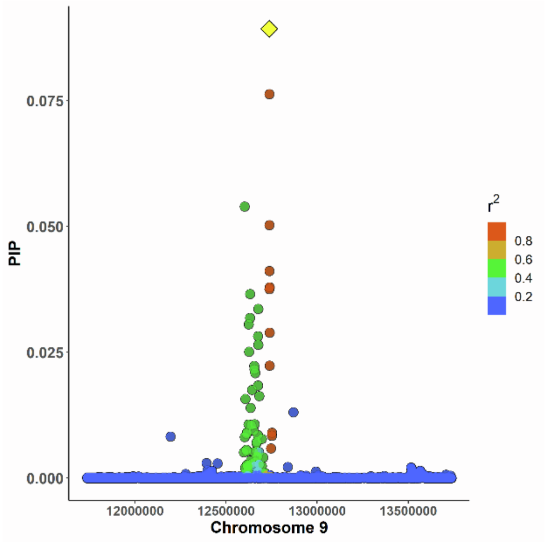

Figure S5. FINEMAP regional plot of the *TYRP1* locus (chromosome 9), Related to STAR Methods. The lead SNP (rs13284327) is highlighted in yellow and LD r^2 correlations are shown with respect to the lead SNP. The candidate causal SNPs in the credible set are located upstream and downstream of the gene.

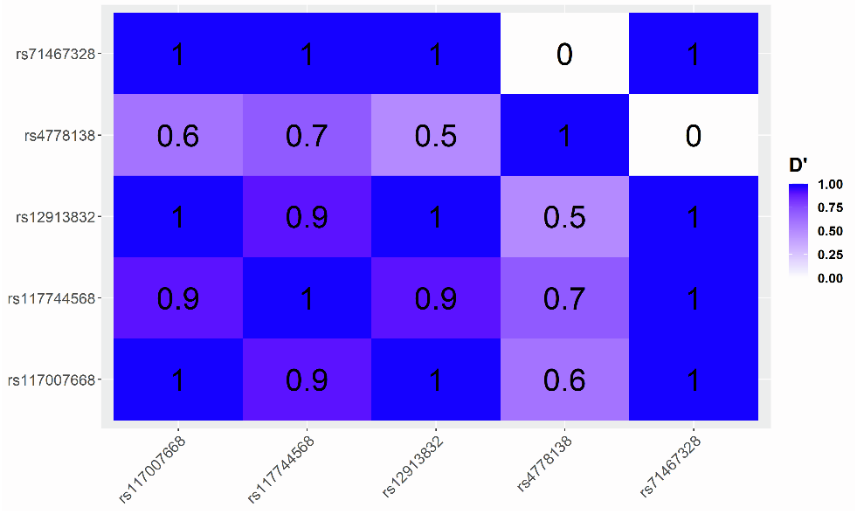

Figure S6. Linkage disequilibrium (D’) among the fine-mapped candidate causal SNPs in the *HERC2/OCA2* locus (chromosome 15) associated with eye colour, Related to Figure 3. D’ was computed on the European subset of individuals from the 1000 Genomes Project on LDLink.

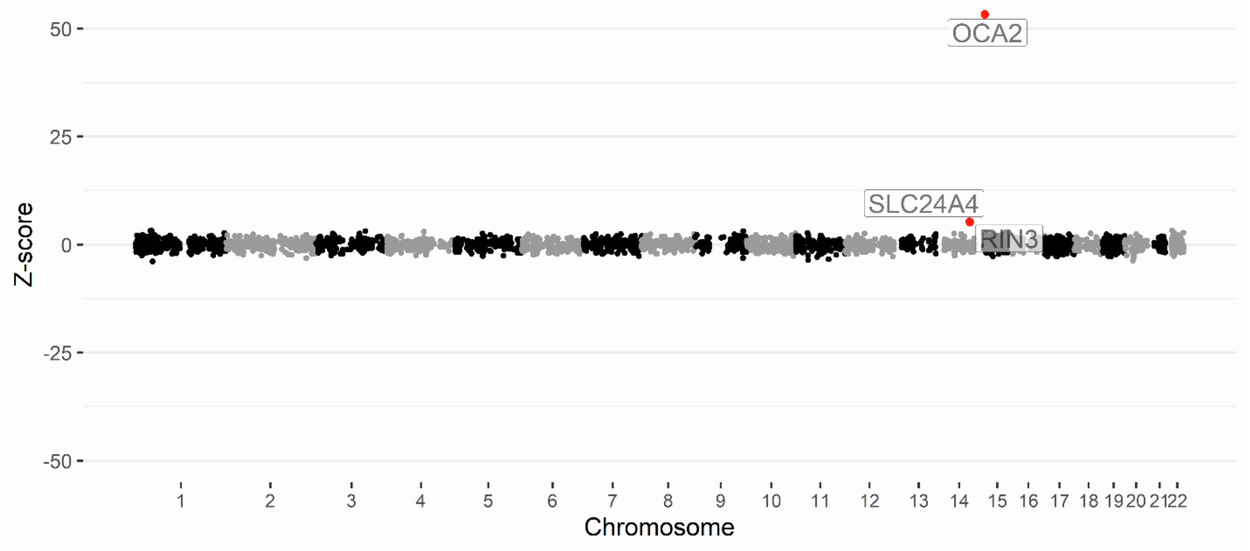

Figure S7. Miami plot of the TWAS conducted for eye colour across chromosomes (X-axis) against TWAS Z-scores (Y-axis), Related to STAR Methods. Genes that passed the significance threshold (p-value ≤ 1.25e-5) are annotated and highlighted in red.

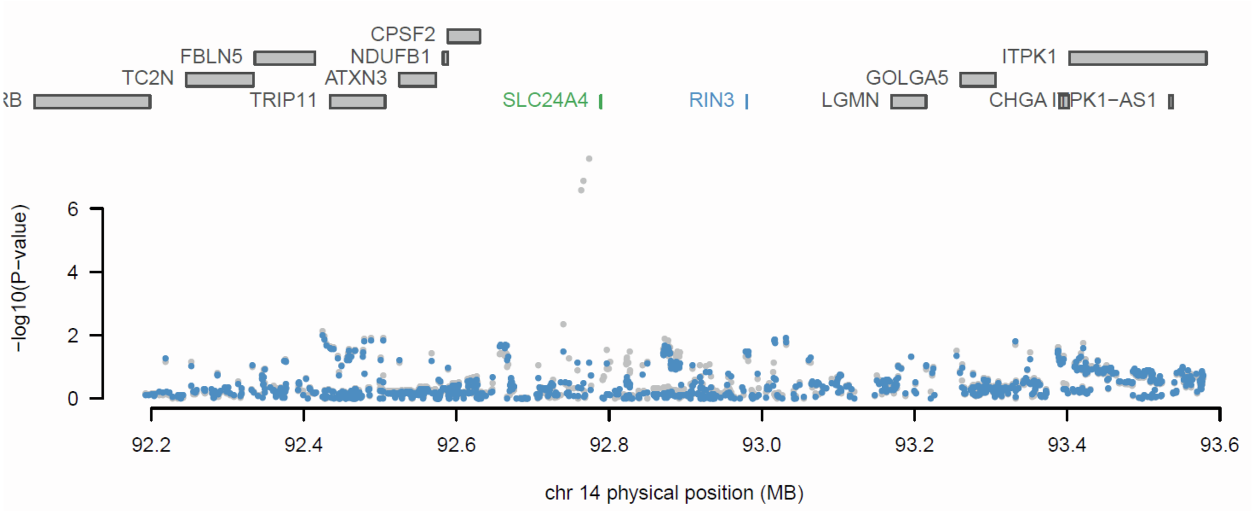

Figure S8. Conditional analysis of TWAS analysis of the locus in chromosome 14, Related to STAR Methods. The top section shows all the genes in the locus: *SLC24A4* and *RIN3*, in blue and green respectively, and other genes are annotated in grey (genome version hg19). The bottom section illustrates the GWAS original regional plot in grey, and after conditioning for the predicted expression of *RIN3* in blue. The GWAS signal disappears after the conditional analysis, indicating that the genes *SLC24A4* and *RIN3* are not independent from each other.

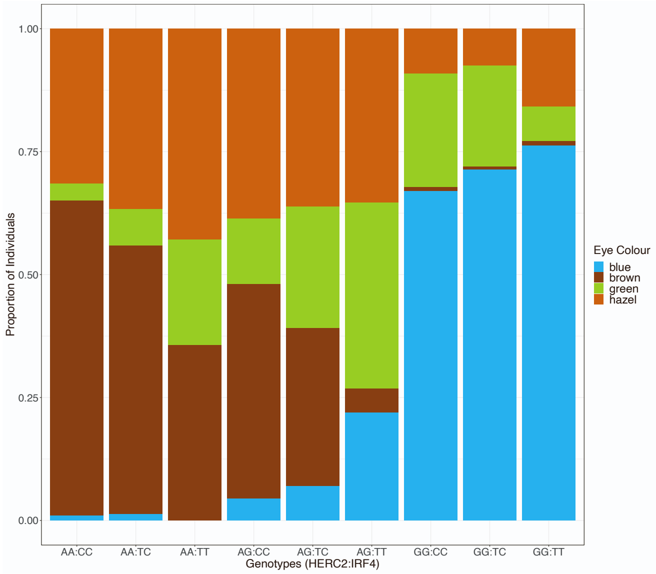

Figure S9. Distribution of eye colour categories in the CanPath cohort, with respect to the *HERC2* rs12913832 and *IRF4* rs12203592 genotypes, showing a negative relationship between the number of rs12203592 T-alleles and the proportion of individuals with brown eye colour, which is significant for the rs12913832-AG genotype when comparing brown vs. non-brown eye colour (Fisher's exact test *p*-value = 2.2e-16) or blue vs. non-blue eye colour (Fisher's exact test *p*-value = 1.22e-07), Related to STAR Methods.
